# Post-transcriptional regulation of erythropoiesis

**DOI:** 10.1097/BS9.0000000000000159

**Published:** 2023-04-26

**Authors:** Yanan Li, Haihang Zhang, Bin Hu, Pan Wang, Wei Wang, Jing Liu

**Affiliations:** a Molecular Biology Research Center, Hunan Province Key Laboratory of Basic and Applied Hematology, School of Life Sciences, Central South University, Changsha, Hunan 410078, China; b Department of Imaging and Interventional Radiology, The Third Xiangya Hospital of Central South University, Changsha, Hunan, China

**Keywords:** Erythropoiesis, mRNA stability, Noncoding RNA, Post-transcriptional regulation

## Abstract

Erythropoiesis is a complex, precise, and lifelong process that is essential for maintaining normal body functions. Its strict regulation is necessary to prevent a variety of blood diseases. Normal erythropoiesis is precisely regulated by an intricate network that involves transcription levels, signal transduction, and various epigenetic modifications. In recent years, research on post-transcriptional levels in erythropoiesis has expanded significantly. The dynamic regulation of splicing transitions is responsible for changes in protein isoform expression that add new functions beneficial for erythropoiesis. RNA-binding proteins adapt the translation of transcripts to the protein requirements of the cell, yielding mRNA with dynamic translation efficiency. Noncoding RNAs, such as microRNAs and lncRNAs, are indispensable for changing the translational efficiency and/or stability of targeted mRNAs to maintain the normal expression of genes related to erythropoiesis. N6-methyladenosine-dependent regulation of mRNA translation plays an important role in maintaining the expression programs of erythroid-related genes and promoting erythroid lineage determination. This review aims to describe our current understanding of the role of post-transcriptional regulation in erythropoiesis and erythroid-associated diseases, and to shed light on the physiological and pathological implications of the post-transcriptional regulation machinery in erythropoiesis. These may help to further enrich our understanding of the regulatory network of erythropoiesis and provide new strategies for the diagnosis and treatment of erythroid-related diseases.

## 1. INTRODUCTION

Erythropoiesis is the process by which hematopoietic stem cells proliferate, differentiate, and mature into red blood cells within the hematopoietic microenvironment.^1,2^ Early erythropoiesis is characterized by the proliferation of erythroid progenitor cells, while late erythropoiesis involves the differentiation and maturation of red blood cells.^3,4^ Burst forming units-erythroid (BFU-E), the earliest progenitor cells committed to erythrocyte maturation, differentiate into colony forming unit-erythroid (CFU-E) during erythropoiesis.^5^ CFU-E then enter terminal erythropoiesis and undergo the successive transition to proerythroblasts (Pro-E), basophilic erythroblasts (Baso-E), polychromatic erythroblasts (Poly-E), and orthochromatic erythroblasts (Ortho-E).^2^ The process of erythropoiesis is accompanied by morphological changes, such as a reduction in cell size, changes in proliferation capacity, as well as the production of hemoglobin.^3^ In the final mature stage, Ortho-E cells expel their nucleus and lose all organelles, forming reticulocytes. These reticulocytes are then released into the blood and continue to mature into fully functional biconcave erythrocytes within 1 to 2 days.^3^

Maintaining a healthy erythropoietic homeostasis is essential for normal body functioning.^6^ If the process is disrupted, it can lead to a variety of blood-related disorders.^6^ According to a report by the World Health Organization, an estimated 30% of the population suffers from anemia, and approximately 8.8% of them are unable to care for themselves because of it, significantly affecting their health and quality of life.^7^ Moreover, an insufficient global blood supply, low erythrocyte expansion in vitro, and low enucleation efficiency are major issues that need to be addressed.^8–10^

Erythropoiesis is precisely regulated by complex networks at transcriptional and post-transcriptional levels, and by signal transduction and epigenetic modifications.^2,9,11,12^ The strict regulation of erythropoiesis is critical for ensuring body homeostasis.^6^ In recent years, research has shifted focus to transcriptional regulation; however, the correlation between protein levels and transcriptional expression is poor, and post-transcriptional regulation/translation is the main determinant of protein abundance in cells.^13^ Therefore, an emphasis on post-transcriptional regulation in erythropoiesis has also emerged in recent years. Post-transcriptional regulation refers to the regulation of gene expression at the post-transcriptional level, reflecting a series of modifications and processing of the transcription products of eukaryotic genes.^13–16^ Post-transcriptional regulation includes the regulation of RNA alternative splicing, m^6^A methylation, regulation of mRNA stability by RNA-binding proteins (RBPs) and regulation of noncoding RNAs, such as microRNA.^13–16^ It has been demonstrated that these post-transcriptional regulatory pathways are vital for normal erythropoiesis, and any abnormalities can lead to erythroid disorder-related diseases.^13–17^

## 2. RNA SPLICING IN ERYTHROPOIESIS

Erythropoiesis uses a complex and comprehensive alternative splicing program to modify gene expression at the post-transcriptional level, ultimately regulating the structure and function of the proteome in a differentiation stage-specific manner.^18^ This program is beneficial for driving differentiation and ensuring the synthesis of the appropriate protein isoforms required for stable red blood cell production.^14,18^

Previous reports demonstrated that RBM38 regulated the activation of protein 4.1R (EPB41) exon 16 during terminal erythropoiesis and was a potent activator of exon 16 splicing.^19^ Recently, the activation of the protein 4.1R exon 16 3’ splice site was shown to require coordination between TIA1, PCBP1, and RBM39 during terminal erythropoiesis.^20^ The mechanism underlying protein 4.1R exon 16 splicing is the binding of TIA1/PCBP1 to the upstream cis elements activating a 3’s through direct interaction with RBM39 and then connecting to SF3b155 for 3’s recognition.^20^ MBNL-binding motifs are enriched near exons undergoing developmental splicing transitions, and MBNL1 is involved in the regulation of the splicing of transcripts (eg, EPB41 and NDEl1).^21^ For example, the knockdown of MBNL1 in cultured murine fetal liver erythroid progenitors disrupts the developmentally regulated exon skipping of NDEL1 mRNA, which is bound by MBNL1 and blocks erythroid terminal proliferation and differentiation.^21^ Similarly, the knockdown of U2AF1 affects the alternative splicing of the gene in erythroblasts. U2AF1 knockdown cells have decreased levels of spliced transcripts encoding MTA1, EIF3B, HNRNPC, and HNRNPD, but increased levels of spliced transcripts encoding BAX and SNHG1.^22^ Erythrocyte gene expression is also regulated by intron retention, where transcripts are polyadenylated and spliced at most introns but remain unspliced at one or more introns.^23,24^ Hundreds of retained introns are dynamically regulated during terminal erythropoiesis, which inversely correlates with expression levels, exhibiting low intron retention in early progenitor cells but higher intron retention in erythroblasts.^23,24^

The mutation of splicing factor SF3B1 disrupts erythroid differentiation via aberrant alternative splicing of transcription factor TAL1. Compared with the SF3B1 wild-type protein, the SF3B1 K700E mutant has stronger binding to the RBP, RBM15, and alters the RNA splicing of the transcription factors TAL1 and GATA1.^25^ Alternative RNA splicing can generate a novel short TAL1 transcript variant (TAL1s). Enhancing the interaction between SF3B1 and RBM15 promotes the production of full-length TAL1 (TAL1fl) mRNA, while reducing the RBM15 protein levels through the PRMT1-mediated degradation pathway which alters the TAL1s/TAL1fl ratio in favor of TAL1s expression.^25^ Because of the structural characteristics of TAL1s, the interaction between TAL1 and ETO2 is blocked, further inhibiting early erythropoiesis.^25^

The dynamic regulation of splicing transitions during erythropoiesis can drive changes in protein isoform expression to add novel functions favorable for erythrocytes. The alternative splicing of the SNRNP70 splicing factor coupled with nonsense mutation-mediated mRNA degradation (NMD) has been reported as a post-transcriptional mechanism downregulating gene expression.^14,23^ Stage-specific splicing of naturally occurring premature termination codons (PTCs) in SNRNP70 exon 8 is low in proerythroblasts; therefore, most transcripts represent translatable mRNA.^14,23^ However, splice transitions convert an increasing proportion of transcripts to NMD-sensitive isoforms in mature cells.^23^

## 3. mRNA STABILITY REGULATION IN ERYTHROPOIESIS

RBPs, key players in post-transcriptional regulation, are involved in regulating the mRNA stability of crucial erythroid factors.^6,13^

**Figure 1.**
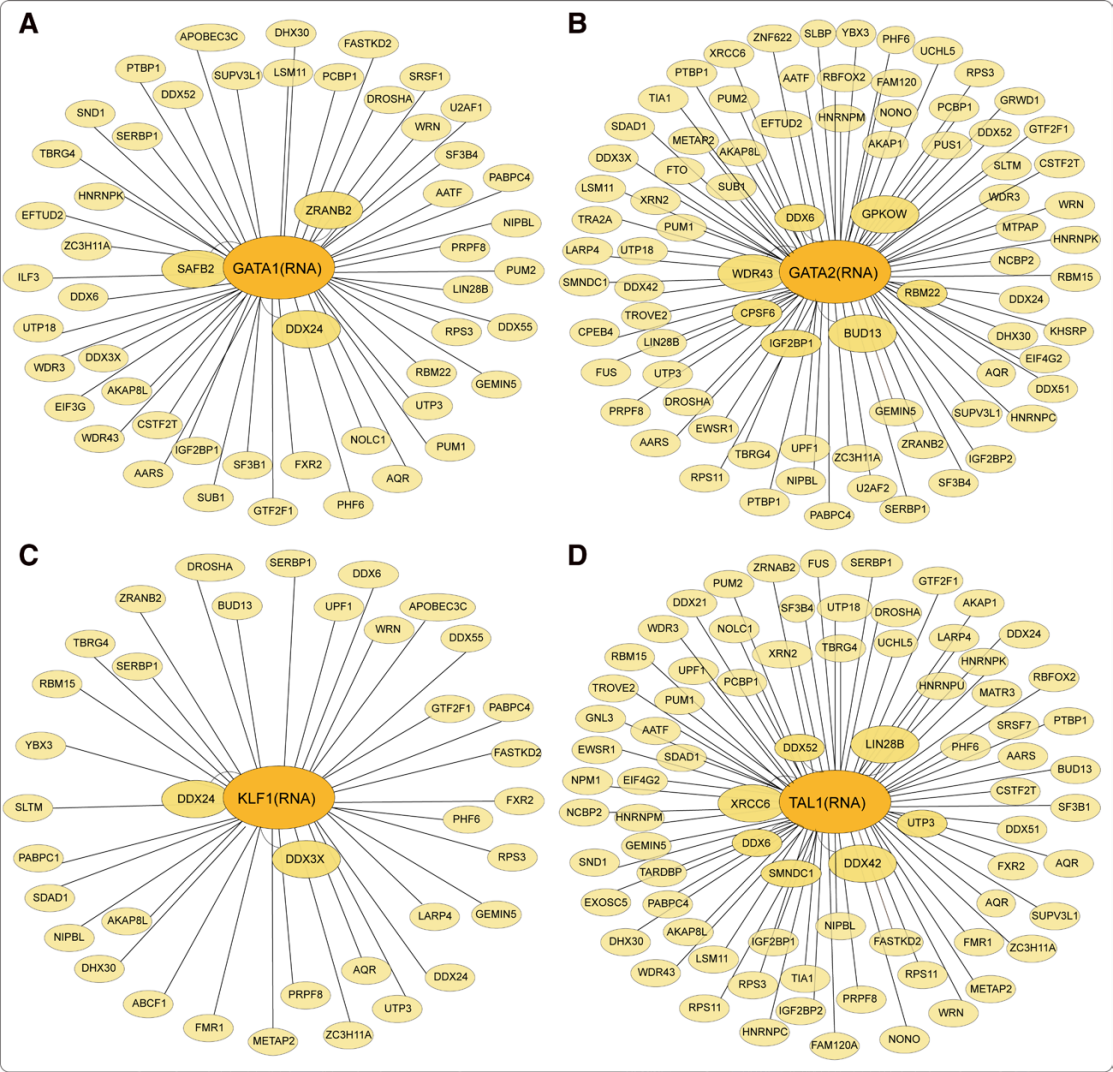
RBPs interaction network of key transcription factors in erythropoiesis (data derived from the POSTAR3 website). RBP = RNA-binding protein.

The normal expression of beta-globin (HBB) protein in mature erythrocytes critically depends on post-transcriptional events in erythroid progenitors that ensure the high stability of HBB mRNA. RBP-PABPC1 has been shown to bind and stabilize HBB mRNA by inhibiting its deadenylation.^26^ Specifically, RBP-AUF-1 and RBP-YB-1 assemble a messenger ribonucleoprotein β-complex on the β-globin 3′ untranslated region (UTR) to enhance the binding of PABPC1 to the poly(A) tail, thus inhibiting mRNA deadenylation and enhancing β-globin mRNA stability in erythroid progenitors.^26^ PABPC4 binds and stabilizes GPA mRNA and other erythroid targets, including HBA1/HBA2, HBB, BTG2, and SLC4A1.^27^ Mechanistically, PABPC4-impacted mRNAs possess a high-value AU-rich motif that enhances the association between PABPC4 and mRNA containing critically shortened poly(A) tails to protect a subset of mRNAs from accelerated decay.^27^ The m^6^A-mRNA-binding protein YTHDF2 can mediate mRNA decay; thus, the activity of these RBPs can antagonize the activity of YTHDF2.^28^ mRNAs with dynamic translation efficiency during erythroid development, whose UTRs are enriched for target sites of specific RNA-binding proteins in hematopoietic cells, regulate the erythrocyte translation program. RBP-RBM38 is specifically induced by GATA1/TAL1 during late erythropoiesis and has been linked to splicing during late erythropoiesis.^19,29^ RBP-RBM38 associates with the translation initiation factor EIF4G and promotes the translation of select mRNAs with decreasing mRNA levels in late differentiating or enucleating cells. The inhibition of RBM38 results in a translation defect and blocks reticulocyte generation, suggesting that RBM38 plays a critical role at the post-transcriptional level during erythropoiesis.^29^ RBP-PCBP1 and PCBP2, which have critical functions in erythropoietic post-transcriptional regulation, modulate the development of erythroid mRNA expression and stability by directly recognizing the cognate poly(C)-rich motif and indirectly mediating alterations in the expression of downstream RNA-binding proteins, such as RBM38.^14,30^ In addition, PCBP2 controls the process of erythropoiesis by regulating the functional splicing of Runx1 transcripts.^31^ The regulatory network of RBPs in erythropoiesis is complex. Gaps exist in our current understanding of the role and mechanism of RBPs in erythropoiesis based on existing reports. Therefore, we obtained the predicted RBP interaction network of erythroid key transcription factors from the POSTAR3 website (**Fig. 1**) to provide unique and helpful insights into the implications of RBP-related regulation in erythropoiesis.

## 4. MICRORNA REGULATION IN ERYTHROPOIESIS

**Figure 2.**
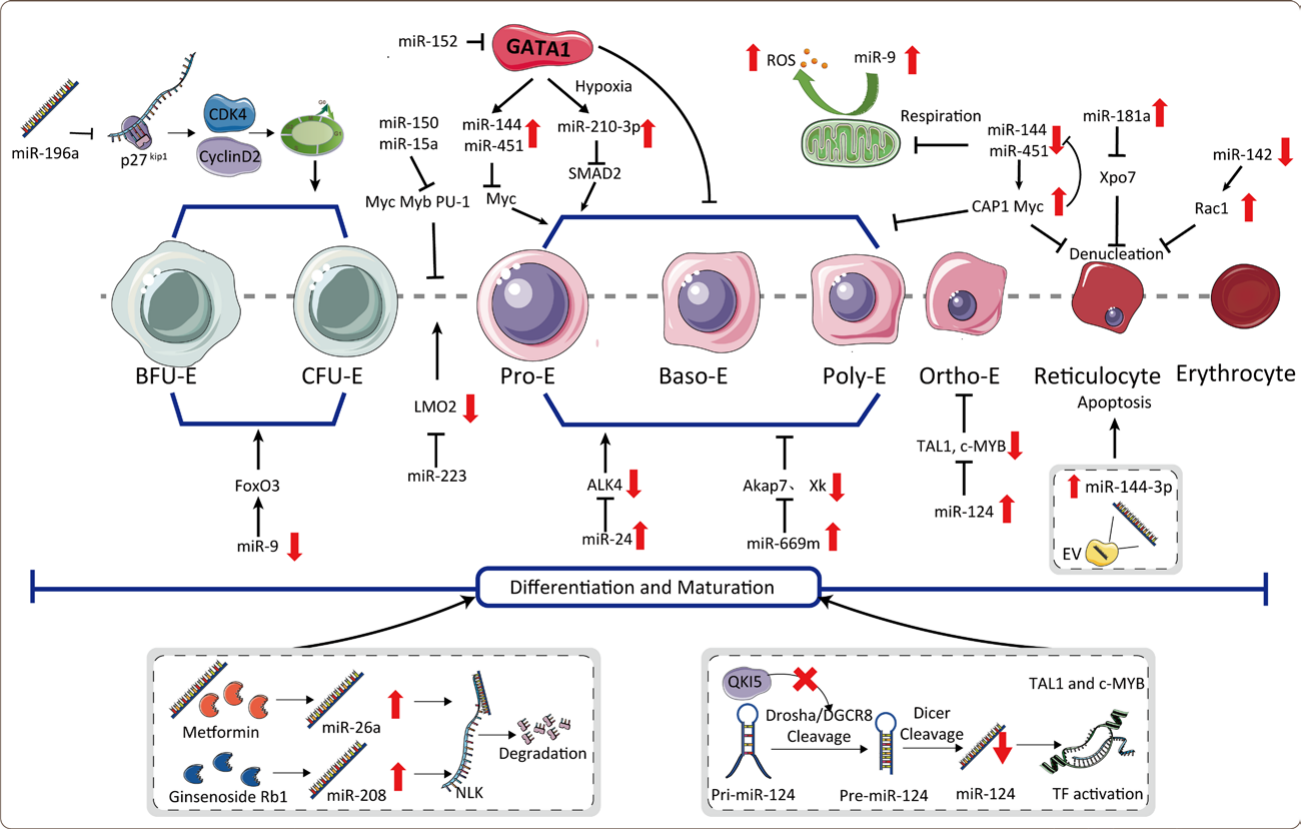
Map of miRNA regulation in erythropoiesis. BFU-E = burst forming units-erythroid, CFU-E, colony forming unit-erythroid, miRNA = microRNA, NLK = Nemo-like kinase, ROS = reactive oxygen species.

MicroRNAs (miRNAs) are small noncoding RNAs that regulate gene expression post-transcriptionally by altering the translational efficiency and/or stability of targeted mRNAs.^32^ MiRNAs, such as miR-142, miR-144, miR-451, miR-221/222, and miR-155, play essential roles in erythroid progenitors, erythroid differentiation, and erythrocyte homeostasis^2,33–54^ (Table 1 and **Fig. 2**).

**Table 1:** Target genes and functions of miRNAs in erythropoiesis.

miRNAs	Target genes	Models/species	Function
miR-124	TAL1, c-MYB	K562 cells	Suppresses red blood cell maturation
miR-142	RAC1	Mice erythroid cells	Maintains steady-state erythropoiesis, hematopoietic progenitor cell proliferation and normal enucleation
miR-144	KLFD, NRF2, RAB14, MEIS1, COX10, MYC, CAP1	Mice fetal liver erythroblasts, MEL cells, and Zebrafish erythroid cells	Negatively regulates oxidative tolerance, mitochondrial respiration and α-globin expression; maintains normal enucleation in erythropoiesis
miR-144-3p	Not applicable (N/A)	Human β-thalassemia erythroid cells	Induces apoptosis
miR-146a	γ-globin	Human β-thalassemia erythroid cells	Inhibits γ-globin expression
miR-152	GATA1	Antarctic icefish	Inhibits erythropoiesis
miR-155	PU-1, ETS-1, CEBP, SHIP1	Mice erythroid cells and K562 cells	Inhibits erythropoiesis
miR-15a/16-1	MYB	K562 cells	Increases γ-globin gene expression
miR-181a	XPO7	Mice erythroid cells	Inhibits red blood cell enucleation
miR-191	RIOK3, MXI1	Mice erythroid cells	Regulates red blood cell enucleation
miR-196a	p27^kip1	K562 cells	Promotes heme-induced proliferation and inhibits apoptosis
miR-200a	PDCD4, THRB	TF-1 cells	Inhibits erythropoiesis
miR-208	NLK	Human Diamond–Blackfan anemia erythroid cells	Maintains normal erythropoiesis
miR-210	HIF, SCF, EPO	K562 cells and β-thalassemia erythroid progenitor cells.	Inhibits γ-globin expression
miR-210-3p	SMAD2	K562 cells	Promotes erythropoiesis under hypoxic conditions
miR-214	ATF4	Thalassemic reticulocytes	Mediates oxidative stress in β-thalassemia disease
miR-221/222	KIT	TF-1 cells	Reduces erythroblast proliferation; regulates hemoglobin switching
miR-222	KIT, BLVRA, CRKL	TF-1 cells and K562 cells	Inhibits erythropoiesis
miR-223	LMO2	K562 cells and human erythroid cells	Regulates erythroid differentiation-related gene expression
miR-2355-5p	KLF6	HUDEP-2 cells and human erythroid cells	Inhibits KLF6 mRNA expression to increase the γ-globin level
miR-24	ALK4	K562 cells and human erythroid cells	Promotes terminal differentiation
miR-26a	NLK	Diamond Blackfan Anemia mice models, human erythroid cells and Zebrafish erythroid cells	Promotes erythropoiesis
miR-27a	CDC25B	K562 cells	Promotes heme-induced erythroid differentiation
miR-320a	SMAR1, TFRC	K562 cells	Inhibits erythrocyte differentiation and apoptosis
miR-326	EKLF	Human erythroid cells	Inhibits HbF synthesis
miR-451	YWHAZ, GATA2, RAB14, GATA1, COX10, c-MYC, CAP1	K562 cells, mice erythroid cells and Zebrafish erythroid cells	Inhibits the nuclear accumulation and mitochondrial respiration of FOXO3; induces γ-globin gene expression; maintains normal enucleation in erythropoiesis
miR-486-3p	BCL11A	Human erythroid cells	Regulates γ-globin expression
miR-486-5p	FOXO1, PTEN	TF-1 cells and human erythroid cells	Controls hematopoietic cell growth and survival
miR-669m	AKAP7, SLC22A4, XK	Mice erythroid cells	Inhibits terminal erythroid differentiation
miR-9	FOXO3	Mice erythroid cells	Blocks erythroid progenitor differentiation; reduces ROS scavenge enzyme expression and increases the ROS level
miR-96	ORF of γ-globin	Human erythroid cells	Inhibits γ-globin expression

HbF = fetal hemoglobin, miRNA = microRNA, ROS = reactive oxygen species.

MiRNAs target negative regulators of erythropoiesis (Table 1).^33–45^ The deletion of miR-142 results in the biconcave shape of red blood cells, impaired structural elasticity, abnormal metabolism of reactive oxygen species (ROS), and shortened overall lifespan.^33,34^ MiR-142 also maintains homeostatic erythropoiesis, hematopoietic progenitor proliferation, and normal enucleation by targeting Rac1 mRNA.^33,34^ Inhibiting the expression of miR-144 and/or miR-451, which is upregulated in erythropoiesis, impairs erythropoietin (EPO)-driven erythropoiesis by targeting RAB14.^36–38^ Recently, miR-144/451 has also been found to block mitochondrial respiration during erythropoiesis by inhibiting the expression of the target gene COX10.^39^ Additionally, CAP1 is a target of miR-144/451 and negatively regulates erythropoiesis and enucleation.^40^ The erythroid-specific transcription factor GATA1 can activate miR-144/451 expression, which directly targets MYC in erythroblasts.^41^ In miR-144/451–depleted erythroblasts, persistently high levels of MYC prevent erythroid differentiation.^41^ Interestingly, Myc inversely regulates the expression of miR-144/451, forming a miR-144/451-MYC positive feedback to ensure a normal level of MYC during erythropoiesis; thus, the GATA1-miR-144/451-MYC network protects normal erythropoiesis.^41^ Moreover, miR-144/451 inhibits the LKB1/AMPK/mTOR pathway to promote the survival of erythrocyte precursors during recovery from acute anemia.^42^

MiRNAs target positive regulators of erythropoiesis (Table 1).^2,46–54^ For example, an elevated expression of miR-155 leads to fewer erythroid progenitors and erythrocytes during erythropoiesis, and exerts its function by targeting the key erythroid transcription factors PU-1 and CEBP.^2,49,50^ MiR-669m is not expressed in erythroblasts, and its overexpression targets AKAP7 and XK to inhibit terminal erythroid differentiation.^47^ The upregulation of miR-152 suppresses GATA1 expression in Antarctic icefish, thereby significantly inhibiting hematopoiesis.^48^ Moreover, QKI5 can activate the processing of primary miR-124-1 (pri-124-1). When erythropoiesis proceeds, decreased QKI5 expression leads to decreased levels of mature miR-124, which increases the expression of the miR-124 target genes TAL1 and c-MYB to promote erythroid maturation.^46^

MiRNAs exert different functions as their expression changes during erythroid development. During the active erythrocyte nuclear transcription stage, a high expression of miR-181a inhibits erythrocyte enucleation by suppressing XPO7 mRNA and protein levels.^55,56^ However, XPO7 expression increases as the miR-181a levels decrease during erythrocyte maturation, thereby condensing the nucleus and promoting enucleation.^55,56^ Heavy ferritin subunit (FHC) silencing leads to an aberrant GATA switch mediated by miR-150 upregulation, thereby blocking erythroid differentiation of K562 cells.^57^

Numerous miRNA analyses based on omics in erythropoiesis have been conducted in recent years. Based on the combined omics analysis of differential miRNA expression, erythroid differentiation gene expression and hemoglobin expression, more downregulated miRNAs than upregulated miRNAs have been found during erythropoiesis, while several lineage-specific miRNAs, such as miR-15b-5p, miR-16-5p, miR-96-5p, and miR-22-3p, were dramatically increased.^58^ In addition, it has been revealed that differences exist in miRNA expression between adult and fetal erythroblasts via omics analyses. There is a predominant expression of let-7 miRNA family members in adult erythrocytes, whereas the chromosome 14q32 miRNA cluster is significantly upregulated in fetal erythroblasts.^59^

## 5. LncRNA REGULATION IN ERYTHROPOIESIS

LncRNAs affect multiple steps of gene post-transcriptional expression, including the regulation of alternative splicing of pre-mRNA, the regulation of mRNA stability and abundance, and the function of competing endogenous RNAs.^60–62^ LncRNAs assist RBPs in functioning as negative regulators of miRNAs in erythropoiesis. It has been shown that the lncRNA, Gm15915, is highly expressed in erythroid progenitor cells and red blood cells. Mechanistically, Gm15915 interacts with WDR82 to promote the transcription of KLF1 and globin genes, thereby regulating the early and late stages of erythropoiesis, respectively, and maintaining erythropoiesis.^63^ The expression of the lncRNA, UCA1, is dynamically regulated during human erythrocyte maturation, with maximum expression in primary erythroid cells, and its deletion primarily impairs heme biosynthesis and prevents erythroid differentiation at the primary erythroid stage.^60^ In terms of mechanism, UCA1 physically interacts with the RBP, PTBP1, and acts as an RNA scaffold to recruit PTBP1 to ALAS2 mRNA, thereby stabilizing it.^60^ Taken together, lncRNA-mediated post-transcriptional mechanisms provide a new dimension for the regulation of erythropoiesis.

## 6. PSEUDOGENE REGULATION IN ERYTHROPOIESIS

It has long been believed that gene duplication drives evolution by generating new genes.^64^ However, most duplicated genes accumulate various loss-of-function mutations during evolution, resulting in pseudogenes.^65^ Since their discovery, pseudogenes have been generally considered nonfunctional. Among various functional mechanisms of pseudogenes, however, antisense transcription-related regulation and competition with miRNA and RBPs appear to be common.^66^

**Figure 3.**
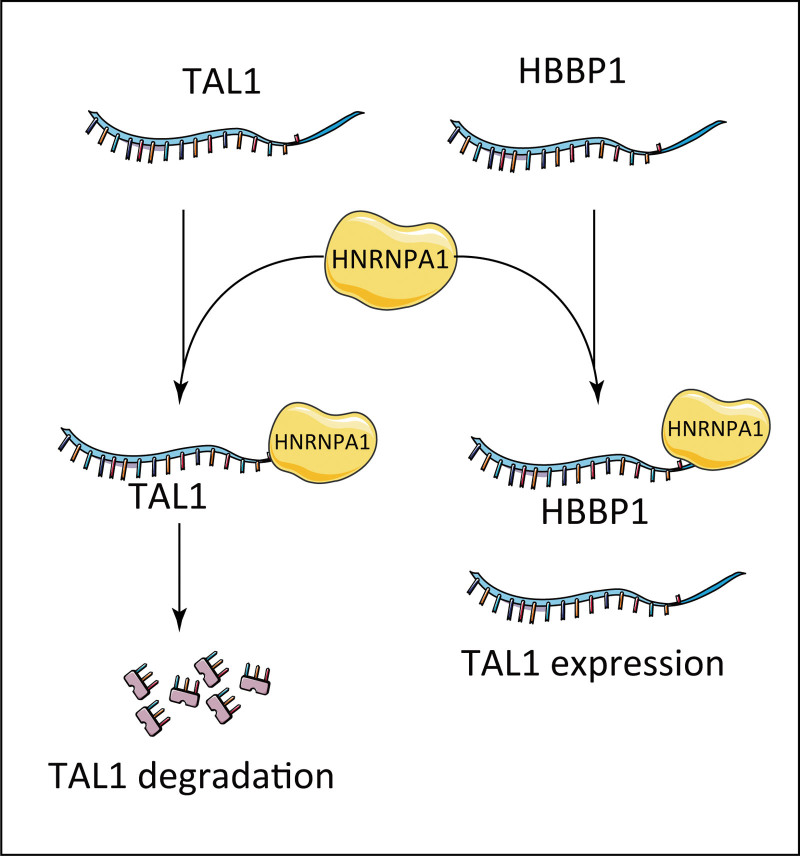
Diagram of the mechanism underlying pseudogene regulation in erythropoiesis. HBBP1=hemoglobin subunit beta pseudogene 1, TAL1=TAL bHLH transcription factor 1, erythroid differentiation factor.

In erythropoiesis, HBBP1, a duplicated pseudogene, has been identified as a key RBP-competing protein for erythroid transcription factors.^65^ Mechanistically, HBBP1 competes with the mRNA of the key transcription factor TAL1 in erythropoiesis to bind the RBP, hnRNPA1, thereby stabilizing TAL1 mRNA, increasing TAL1 protein expression and maintaining normal erythropoiesis^65^ (**Fig. 3**). HBBP1 deletion leads to the complete loss of the ability of embryonic stem cells to differentiate into erythroid cells and inhibits the differentiation process of hematopoietic stem/progenitor cells in vivo and in vitro.^65^

## 7. N6-METHYLADENOSINE METHYLATION REGULATION IN ERYTHROPOIESIS

More than 60% of RNA modifications have been shown to involve methylation, and m^6^A is the most common modification of mRNA, lncRNA and various other noncoding RNAs in higher organisms.^67^ The function of m^6^A modification is mainly determined by “encoder (writer, namely, base transferase),” “decoder (eraser, namely, demethylase),” and “reader (reader, namely, m^6^A binding protein).”^68^ The known methyltransferase complex components are METTL3, METTL14, WTAP, and KIAA1429. The demethylases that reverse methylation include ALKBH5 and FTO, and the m^6^A-binding proteins include the YTH domain protein and the nuclear heterogeneity protein HNRNP family.^69^

Recently, the role of m^6^A-dependent regulation of mRNA translation in maintaining the erythrocyte gene expression program and promoting erythroid lineage decisions has been clarified. We found that three core components of the m^6^A MTase complex (METTL14, METTL3, and WTAP) are required for the maintenance of GPA expression in HEL cells and erythroid lineage determination in human stem/progenitor cells. Moreover, m^6^A MTase activity is mediated by the selective translation of approximately 300 m6A-tagged mRNAs to promote the erythrocyte gene expression program.^15^ The important regulatory role of m^6^A RNA methylation in erythropoiesis has also been demonstrated by CRISRP-Cas9 comprehensive screening.^70^ With regard to the physiological role of the m^6^A-dependent regulation of mRNA translation in maintaining the erythrocyte gene expression program and promoting erythrocyte lineage determination, it was found that the inhibition of the methyltransferase complex-mediated loss of m^6^A resulted in a disruption of the erythrocyte transcriptional program; this did not directly inhibit previously identified master transcriptional regulators (eg, GATA1 and KLF1) but resulted in a variety of other gene translation downregulations.^15^ Furthermore, the maintenance of the erythroid transcriptional program is partially driven by the m^6^A translational regulation of the SETD1A/B complex, which promotes the recruitment of the transcriptional activation markers H3K4me3 and KLF1 transcription factors to erythroid gene promoters.^15^

## 8. DISEASES RELATED TO GENE POST-TRANSCRIPTIONAL DYSREGULATION IN ERYTHROPOIESIS

Maintaining normal erythropoiesis relies on the tight and precise regulation of erythroid development in healthy individuals.^6^ In ineffective erythropoiesis, there is an imbalance due to an increased proliferation of erythroid progenitors accompanied by increased apoptosis and decreased erythrocyte maturation.^71,72^ Ineffective erythropoiesis leads to a series of anemia-related blood diseases, including myelodysplastic syndrome (MDS), β-thalassemia (β-TM), and Diamond-Blackfan anemia (DBA).^72–74^

Ineffective erythropoiesis, erythroid hyperplasia, and anemia phenotypes have been reported in the miR-142-/- mouse model. MiR-451/144 deficiency leads to erythroid hyperplasia and mild anemia, whereas miRNAs (eg, miR-150, miR-223, miR-15a, and miR-24) are upregulated in individuals with persistent injury, thereby inhibiting erythropoiesis and resulting in the development of persistent injury-related anemia.^75^ Therefore, miRNA oligonucleotides or anti-miRNA oligonucleotides may serve as potential therapeutic strategies for the treatment of erythroid anemia-related diseases. We summarize here some potential links to the post-transcriptional regulation of erythropoiesis and related treatment strategies for MDS, β-TM, and DBA.

### 8.1. Myelodysplastic syndrome

MDS is a clonal blood disorder characterized by abnormal hematopoietic development and cytopenia that is usually caused by mis-sense mutations in genes encoding the splicing factors SF3B1, ZRSR2, U2AF1, U2AF2, or SRSF2.^76–81^ The most common spliceosome mutations observed in MDS include U2AF1, with mutation hotspots in the zinc finger domains at codons S34 and R156/Q157; SF3B1, with hotspots, such as K700 and K666 in the HEAT domains; and SRSF2, with a hotspot at codon Pro95.^81^ The mutant protein exhibits relatively modest changes in RNA-binding specificity; however, such changes are sufficient to induce aberrant 3′ splice site selection in transcripts in many patients.^77^ SF3B1 mutations are among the most common events, and in the case of SF3B1 mutants, branch point recognition is altered and can promote aberrant splicing.^78,82,83^ ABCB7 expression is reduced in MDS patients with SF3B1 mutation owing to splicing of the aberrant 3′ splice site that alters the translational reading frame, introduces PTC and activates NMD.^84^ Studies have demonstrated that the physiologic expression of SF3B1 ^K700E^ mutation causes impaired terminal erythropoiesis, erythroid dysplasia, aberrant splicing and sensitivity to pharmacologic spliceosome modulation, which is sufficient to cause the characteristic features of MDS.^78,85^ K562 cells with the SF3B1 ^K700E^ mutation exhibit accelerated differentiation and increased erythroid-cell death, which is due to mis-spliced MAP3K7 transcripts leading to specifically deregulated p38 MAPK.^86^ SF3B1 deficiency impairs human erythropoiesis by decreasing the expression of the large MKRN1 isoform to activate the p53 pathway; this improves our understanding of ineffective erythropoiesis in MDS patients with SF3B1 mutations.^87^ SRSF2 Pro95 hot spot mutations elicit enhanced mRNA decay, which depends on sequence-specific RNA binding and splicing.^80^ SRSF2 mutants enhance the deposition of exon junction complexes (EJCs) downstream of the PTC through RNA-mediated molecular interactions, whose architecture favors the association of key NMD factors to elicit mRNA decay.^80^

In addition, m^6^A mRNA methylation was detected in 70 of 104 known MDS genes, including 8 of the 10 most commonly mutated genes (eg, RPS19, TET2, SF3B1, ASXL1, RUNX1, DNMT3A, ZRSR2, and STAG2).^15^ The U2AF1 ^S34F^ mutation exhibits lineage specificity in altering the pre-mRNA splicing of downstream target genes, resulting in distinct phenotypes in different myeloid lineages involved in MDS.^88^ Therefore, the study of post-transcriptional regulation in erythropoiesis is potentially important for understanding MDS and developing corresponding therapeutic strategies.

### 8.2. β-thalassemia

β-TM is a type of β-hemoglobinopathy known for clinical heterogeneity in which patients exhibit elevated fetal hemoglobin (HbF) levels.^89^ Based on combined omics analyses of differential miRNA expression, erythroid differentiation gene expression and hemoglobin expression, miRNAs and their associated genes or pathways could be explored as potential targets for new therapies for β-hemoglobinopathies and other erythropoietic disorders.^58^ During erythroid differentiation, more miRNAs are downregulated, while the expression of several lineage-specific miRNAs (eg, miR-15b-5p, miR-16-5p, miR-96-5p, and miR-22-3p) is dramatically increased in the terminal stages.^58^ It has been reported that in β-TM, 8 miRNAs (hsa-miR-146a-5p, hsa-miR-146b-5p, hsa-miR-148b-3p, hsa-miR-155-5p, hsa-miR-192-5p, hsa-miR-335-5p, hsa-miR-7-5p, and hsa-miR-98-5p) were identified as significantly upregulated, while 4 miRNAs (hsa-let-7a-5p, hsa-miR-320a, hsa-let-7b-5p, and hsa-miR-92a-3p) were significantly downregulated.^11^ Further analysis revealed that these miRNAs are related to a variety of biological processes and molecular functions, including the MAPK and HIF-1 signaling pathways related to the upregulation of HbF.^11^ Therefore, the high level of HbF in β-TM individuals may be mediated by miRNAs. The upregulation of miR-214 mediates oxidative stress in β-TM by targeting ATF4.^90^ Significantly increased levels of extracellular vesicle miR-144-3p were observed in β-TM and induced apoptosis, which may lead to organ dysfunction and complications in patients with β-TM.^91^ MiR-2355p increases the γ-globin levels by targeting KLF6 mRNA to promote normal cell expansion in β-TM patients, providing more information for the clinical management of patients.^35^ In individuals with severe β-TM, the increased miR-326 expression in reticulocytes was positively correlated with HbF levels.^52^ Therefore, miR-326 may play an important role in regulating globin turnover during β-TM stress-induced erythropoiesis.^52^ In plasma, miR-451 acts as a novel hemolytic marker for β-TM/hemoglobin E (HbE) disease.^92^

HBBP1 competes with TAL1 mRNA, a transcription factor critical for erythroid development, to bind the RBP, hnRNPA1, thereby improving the stability of TAL1 mRNA and maintaining normal erythropoiesis.^65^ HBBP1 also has an important protective effect in patients with β-TM. HBBP1 is compensatively increased in β-TM patients and participates in HbF activation and anemia symptom relief in patients by promoting the stable expression of TAL1.^65^

Additionally, point mutations within exons or their flanking introns interfere with the splicing of many erythroid transcripts. Owing to changes in exon skipping or splice site selection that alter the translation reading frame, introduce premature stop codons and activate NMD, splice mutants often manifest as underexpressed alleles, a common cause of β-TM and erythrocyte membrane disorder.^14^

### 8.3. Diamond-Blackfan anemia

Disturbing the isoform balance between alternatively spliced transcripts may contribute to the development of DBA. Partial skipping of exon 2 in the GATA1 gene occurs naturally to generate transcripts encoding shortened protein isoforms called GATA1s, and the deficiency of full-length GATA1 affects erythropoiesis.^93^ In DBA patients, mutations in the 3′ end splice site of exon 2 in the GATA1 gene were found to be almost eliminated.^93^

In addition, miRNA agonists or inducers represent a potential new approach for DBA therapy. It has been reported that metformin induces the expression of miR-26a and recognizes the binding site within the 3′-UTR of Nemo-like kinase (NLK) to promote the degradation of NLK transcripts, thereby promoting erythroid differentiation and improving anemia in DBA patients.^94^ Similarly, ginsenoside Rb1 upregulates the expression of miR-208, which binds the 3′-UTR of NLK mRNA and targets it for degradation, thereby improving abnormal erythropoiesis in DBA patients.^95^

### 8.4. Myeloproliferative neoplasms

Myeloproliferative neoplasm (MPNs), such as polycythemia vera (PV), essential thrombocythemia (ET), and primary myelofibrosis (PMF), are a heterogeneous group of clonal hematopoietic disorders characterized by myeloid progenitor proliferation, which involve an excess of differentiated erythrocytes, platelets, and leukocytes circulating in peripheral blood.^96^

RNA splicing factor mutation is one of the most frequent mutations in MPN. The SF3B1 mutation occurs in 5% to 10% of MPN patients. The SRSF2 mutation affected transcriptional regulation through predominant splicing of RUNX1 to form the RUNX1a transcript.^97^ The short isoform RUNX1a overexpression has been reported in MPN.^98^ The SRSF2 mutation is found in 3% to 20% of MPN, with lower frequencies in PV and ET compared with PMF.^99^ In PMF, U2AF1 mutations were associated with inferior survival.^100^ PMF, PV, and ET patients with U2AF1 mutations have a poorer myelofibrosis-free survival compared with wild-type patients. A total of 65% of U2AF1 mutations affect Q157 and the presence of this mutation is associated with significantly shorter overall survival in MPNs.^101^

The application of RNA splicing factor mutation in the early risk stratification of patients increases the possibility of early intervention to prevent the progression of MPN. However, more research is needed to explain the potential molecular mechanisms and effectively target them.

## 9. CONCLUSIONS AND PERSPECTIVES

The importance of erythropoiesis for normal human body function underscores the need for multilevel research. Dynamic regulation of splicing transitions during erythropoiesis leads to changes in protein isoform expression that add new functions beneficial for erythropoiesis. RNA-binding proteins adapt the translation of transcripts to the protein requirements of the cell, resulting in mRNA with dynamic translation efficiency. The m^6^A-dependent regulation of mRNA translation plays an important role in maintaining erythrocyte gene expression programs and promoting erythroid lineage determination. Noncoding RNAs, such as miRNAs and lncRNAs, are indispensable for maintaining the normal expression of genes related to erythropoiesis and targeted therapy for abnormal erythroid diseases.

**Figure 4.**
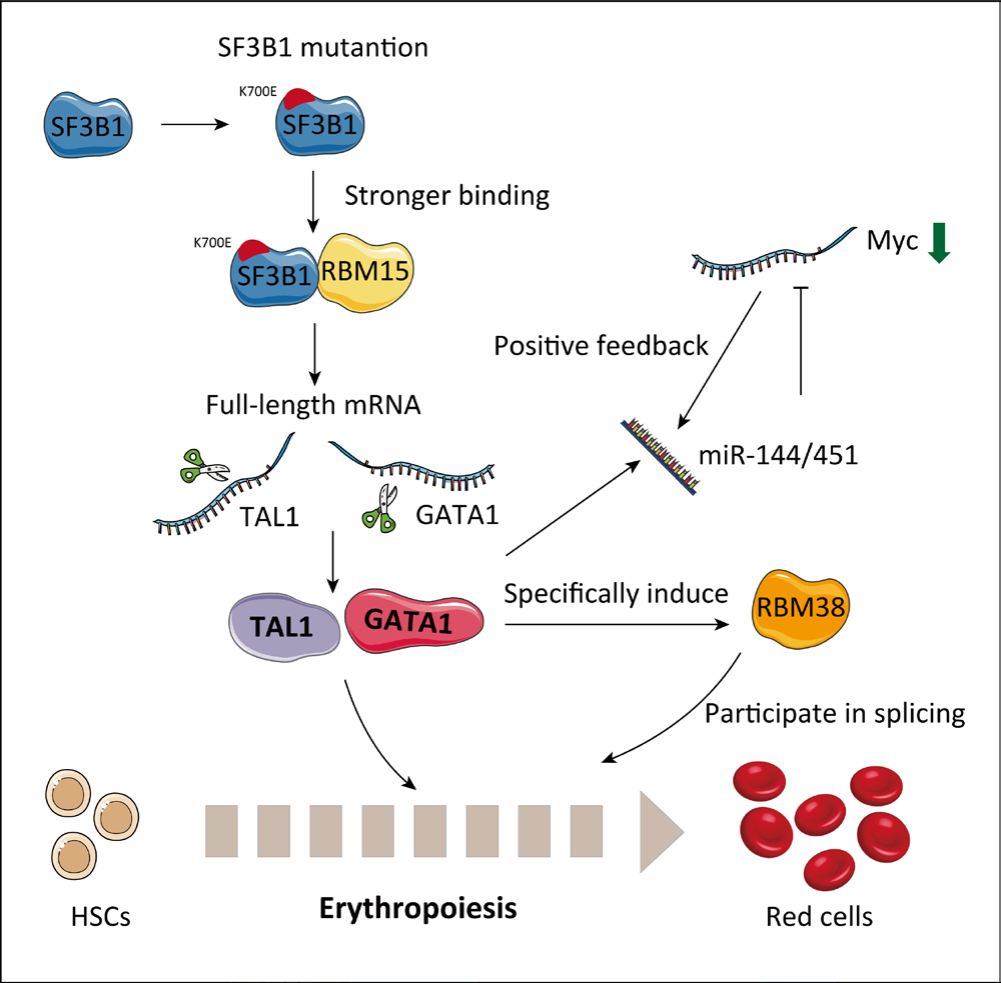
Diagram of post-transcriptional regulatory network centered on GATA1 and TAL1. HSC = hemapietic stem cell.

In this review, we have summarized recent research in the post-transcriptional regulation of erythropoiesis. These post-transcriptional regulatory patterns can form regulatory networks centered on certain genes that are critical for erythropoiesis, thus controlling the whole process of red blood cell development (**Fig. 4**). In some instances, splicing factor mutations as exemplified by SF3B1 and U2AF1 have provided diagnostic utility and information on clinical outcomes for erythropoiesis-related diseases, such as myelodysplastic syndrome and MPNs. However, more research is needed to explain the potential molecular mechanisms and effectively target them, so as to achieve the purpose of clinical treatment. Although miRNAs and RNA-binding proteins involved in post-transcriptional regulation are key players in erythropoiesis, their specific roles of these particular pathways in erythroid development remain largely unknown, given the relative infancy of the miRNA/RNA-binding protein field. In addition, the post-transcriptional regulatory network of m^6^A during erythropoiesis requires further elucidation. Based on our understanding of the existing post-transcriptional regulation mechanism during erythropoiesis, abnormal post-transcriptional regulation during erythropoiesis can lead to a variety of ineffective erythropoiesis-related diseases, such as myelodysplastic syndrome, β-thalassemia, and Diamond-Blackfan anemia. Consequently, further exploration and discovery of new post-transcriptional regulatory mechanisms will enrich our understanding of the regulatory network of erythropoiesis and provide new strategies for the diagnosis and treatment of erythroid-related diseases.

## ACKNOWLEDGMENTS

This work was supported by grants from the National Natural Science Foundation of China (Grant numbers 81920108004 and 82270127); the Fundamental Research Funds of the Central Universities of Central South University (Grant number 2021zzts0562); and the Fundamental Research Funds for the Scientific Research Innovation Project of Hunan Province (Grant number CX20210182).

## AUTHOR CONTRIBUTIONS

J.L. designed this study. Y.L. drafted the manuscript. H.Z., B.H., and P.W. collected the data. J.L. and W.W. revised the manuscript. All authors have read and approved the final manuscript.
